# A Rare and Challenging Case of Refractory Fetal Supraventricular Tachycardia

**DOI:** 10.7759/cureus.28947

**Published:** 2022-09-08

**Authors:** Lindsay Celentano, Kai Yoshinaga, Steven K Shiba, Zachary Gaynor, Jane Rudolph

**Affiliations:** 1 Obstetrics and Gynecology, Charles E. Schmidt College of Medicine, Florida Atlantic University, Boca Raton, USA; 2 Internal Medicine, Charles E. Schmidt College of Medicine, Florida Atlantic University, Boca Raton, USA; 3 Obstetrics and Gynecology, Nova Southeastern University Dr. Kiran C. Patel College of Osteopathic Medicine, Davie, USA; 4 Obstetrics and Gynecology, Boca Raton Regional Hospital, Boca Raton, USA

**Keywords:** atrio-ventricular re-entrant tachycardia, fetal therapeutics, maternal fetal complications, cordocentesis, cardiac electrophysiology, fetal echocardiogram, supra ventricular tachycardia, wolff-parkinson-white

## Abstract

Fetal supraventricular tachycardia can be difficult to manage and offers a challenging treatment course, particularly in refractory cases. The treatment course must balance maternal well-being with the health status of the fetus, all while racing against possible progression to hydrops fetalis or permanent cardiac dysfunction. We describe a case of fetal supraventricular tachycardia that demonstrates many of these concepts, as well as the importance of utilizing several treatment pathways in refractory cases.

## Introduction

Fetal tachyarrhythmias are present in 0.4%-0.6% of all pregnancies, with supraventricular tachycardia (SVT) being the most common form of fetal tachycardia [1,2]. Of patients with fetal SVT, approximately 5% can be attributed to a structural cause [3]. More common mechanisms include atrial flutter or atrioventricular re-entry tachycardia (AVRT). Wolff-Parkinson-White (WPW) syndrome is an example of AVRT which involves an abnormal alternate electrical pathway between the atria and ventricles [4]. In addition to causing supraventricular tachyarrhythmias, WPW can lead to cardiac arrest and even sudden cardiac death; therefore, early diagnosis and treatment is essential. WPW has an incidence of 0.1%-0.2% among neonates, and up to 37% of infants diagnosed with WPW also have a congenital cardiac defect [5].

While data is not conclusive, around 41% of fetal SVT cases will progress to hydrops fetalis [6,7]. First-line treatment of fetal SVT remains controversial because most studies are observational or retrospective, and they are often confounded by physician preference [8,9]. The protocols that do exist typically involve antiarrhythmics such as sotalol, digoxin, and flecainide [2,7-10]. There is also an ongoing randomized controlled trial seeking to provide clarity to which agents are preferred [11]. Careful monitoring during transplacental treatment is necessary because serious fetal and maternal adverse events may occur, particularly in cases when combination treatment is needed [12]. Unfortunately, greater than 50% of patients require multiple antiarrhythmic agents due to refractory disease, a term that is not uniformly defined in the literature [13]. For example, in a 2017 multicenter study, cases of SVT were considered refractory if first-line therapy failed [13]. Another report considered cases to be refractory if there was no improvement or if deterioration of fetal status was seen despite therapeutic levels [14]. Due to the critical nature of refractory cases and the lack of consensus for treatment regimen, there should be strong familiarity with treatment options for refractory cases to ensure patients are managed appropriately and quickly enough to avoid morbidity and mortality. We present the case of a 27-year-old woman at 32 weeks whose fetus was found to have SVT refractory to several treatments.

## Case presentation

The patient is a 27-year-old female G4P2 with a history of epilepsy controlled on lamotrigine. At 32 weeks and 0 days, she presented to her perinatologist, who she was originally referred to due to her history of epilepsy, for a follow-up appointment. At this visit, the fetus was incidentally found to have tachycardia of 220-240 bpm with reactivity (at least two accelerations in a 20-minute period that peak 15 beats per minute or more above baseline and that last for at least 15 seconds) and moderate variability (fluctuations in fetal heart rate between six and 25 beats per minute from baseline). Additionally, the fetus was found to have a pericardial effusion visualized on echocardiogram, though hydrops fetalis was not detected.

The patient was admitted to the hospital where she was initially treated with sotalol 80 mg three times a day by mouth for two days, followed by four days of continued sotalol in combination with digoxin 250 mcg daily by mouth. On hospital day 7, cordocentesis with a 7.3 mg bolus of adenosine was attempted three times during one procedure without success. On hospital day 8, the patient was administered flecainide 100 mg three times daily by mouth in combination with continued sotalol at the same dose as a last resort while preparing for the induction. The fetus cardioverted spontaneously after the fourth dose of flecainide. The fetal heart rate stabilized at 130-140 bpm with moderate variability. Given the improvement in fetal status and decreased risk of progression to hydrops fetalis, the induction was deferred to 37 weeks barring further complications.

**Figure 1:**
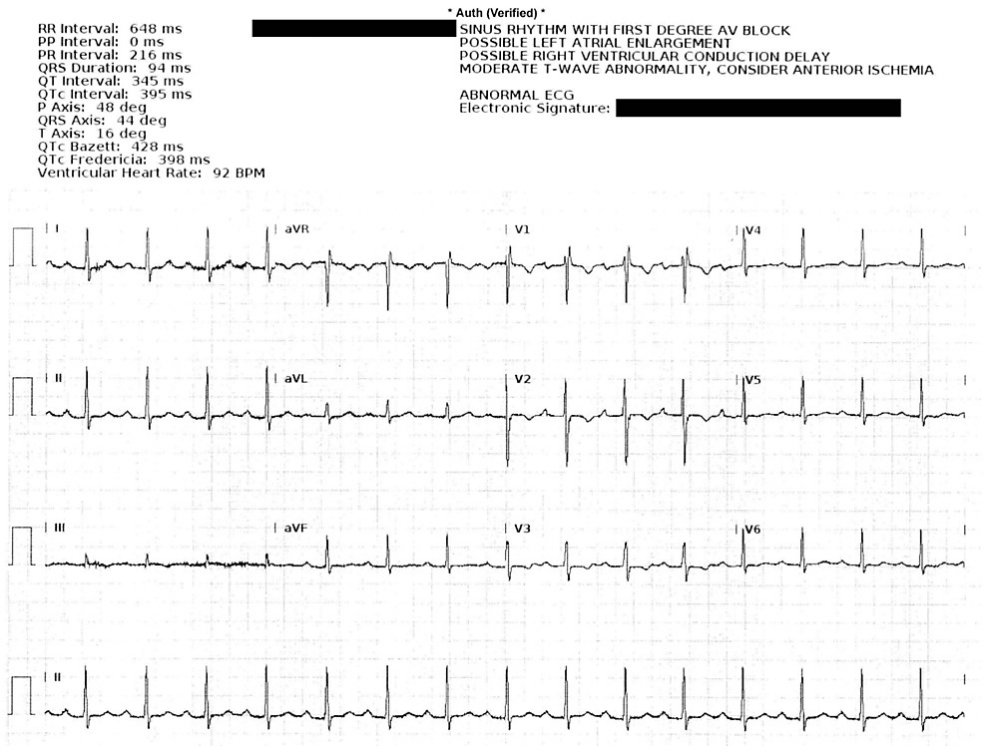
Maternal ECG on admission. Note prolonged PR interval.

**Figure 2:**
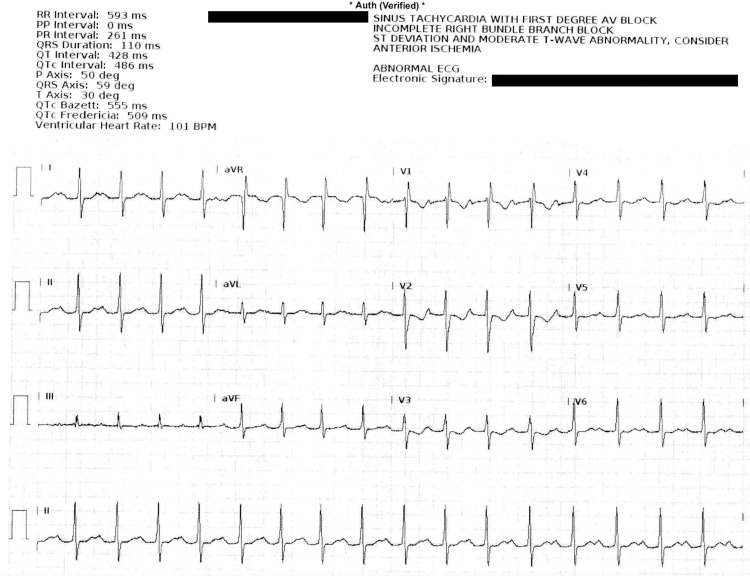
Maternal ECG on hospital day 9. Note widened PR, QRS, and QTc intervals.

On hospital day 9, the consulting electrophysiologists on the case noted that the patient’s QRS, QTc, and PR intervals were lengthened from baseline, leading to concerns about progression to torsade de pointes. Note that on admission the patient’s ECG showed a baseline of first-degree AV block (see Figure 1 and Figure 2 for admission and day 9 ECGs, respectively). The next day the sotalol was discontinued and by hospital day 14 the intervals returned to the patient’s baseline while the fetal heart rate remained stable.

The patient was discharged on the same dose of flecainide and was provided a Holter monitor for outpatient monitoring. In the following weeks, the patient attended weekly scheduled appointments with her obstetrician, perinatologist, and electrophysiologist. During this time the maternal ECG and the fetal heart rate remained stable and there were no concerning findings on sonographic examinations. The patient was readmitted three weeks later for scheduled induction of labor using oxytocin, and vaginal delivery was performed without complications. The female neonate was born at 36 weeks and 6 days with APGAR scores of 9 and 9 at one and five minutes, respectively. The maternal postpartum course was uncomplicated. The neonate, who was previously receiving SVT control via maternal ingestion of flecainide, required five days of NICU observation with increasing dosages of propranolol, escalating from 1 mg of propranolol daily to 5.85 mg daily, to suppress recurrences of her SVT. The underlying etiology of the infant's SVT was not initially elucidated. However, due to her stability and maintenance of sinus rhythm, the infant was discharged on 2 mg propranolol twice daily with continuous fetal heart rate monitoring.

Outcome and follow-up

On evaluation in the pediatrician’s office two days post discharge, the neonate was healthy and there were no immediate concerns. Five days later she was evaluated in a pediatric cardiologist’s office and was found to have prolonged QRS with delta waves. Given the presentation, Wolff-Parkinson-White syndrome was diagnosed. Management included oral propranolol with a plan for eventual ablation at eight years of age or when she reached 15 kg, depending on electrophysiologist evaluation later in life.

## Discussion

This case demonstrates some of the difficulties associated with managing fetal SVT, balancing maternal health with management needs, and the many treatment pathways that refractory cases may require before success. AVRT, antidromic or orthodromic, is typically the presenting arrhythmia of WPW. Clinical manifestations of WPW can be recognized in utero, however, most cases are not diagnosed until early childhood [5]. A fetus may present with episodes of SVT which can result in hydrops fetalis depending on the duration and severity of the tachyarrhythmia. ECG is the primary diagnostic tool for WPW in neonates. Echocardiogram can be performed to determine the presence of a concurrent anatomical defect [15].

Treatment of fetal SVT and fetal WPW varies broadly between groups and is often provider dependent. Patient presentation, age, and presence of a congenital defect are all factors that must be considered. In utero, management strategies are restricted to pharmacologic therapy that is administered to the pregnant mother and acts on the fetus transplacentally. Post delivery, management strategies can also include vagal stimulation, catheter ablation, and ablation during open heart surgery. Pharmacologic treatments are tailored to the patient and regimens, which may involve single or multiple agents, vary widely [8-10]. For example, in our case the progression of medication was as follows: sotalol alone, followed by sotalol in combination with digoxin, followed by cordocentesis with direct administration of adenosine, followed by flecainide with sotalol, followed by flecainide alone. Even the eventual successful treatment regimen required adjustment to bring the patient to 37 weeks gestation, as the mother's QRS and PR interval lengthening on sotalol was disconcerting. This case demonstrates the importance of having strong familiarity with treatment pathways for refractory cases to ensure patients are managed appropriately and quickly enough to avoid morbidity and mortality.

## Conclusions

Fetal SVT and WPW are potentially morbid and fatal complications of pregnancy that demand dynamic and multimodal treatment strategies to optimize care and reduce risk to both the mother and fetus. This challenging case illustrates the potential strategies and difficulties in managing these patients, especially when refractory.
